# A digital intake tool to avert outpatient visits in a FIT-based colorectal cancer screening population: study protocol of a multicentre, prospective non-randomized trial - the DIT-trial

**DOI:** 10.1186/s12876-023-03039-0

**Published:** 2024-01-18

**Authors:** Fleur E. Marijnissen, Pieter J. F. de Jonge, Nicole S. Erler, Sohal Y. Ismail, Iris Lansdorp-Vogelaar, Manon C. W. Spaander

**Affiliations:** 1 Department of Gastroenterology and Hepatology, Erasmus MC, University Medical Center, Rotterdam, the Netherlands; 2 Department of Biostatistics, Erasmus MC, University Medical Center, Rotterdam, the Netherlands; 3 Department of Epidemiology, Erasmus MC, University Medical Center, Rotterdam, the Netherlands; 4 Department of Psychiatry, Section Medical Psychology and Psychotherapy, Erasmus MC, University Medical Center, Rotterdam, the Netherlands; 5 Department of Public Health, Erasmus MC, University Medical Center, Rotterdam, the Netherlands

**Keywords:** Colorectal cancer screening, Telemedicine, Patient education, Counselling, Colonoscopy

## Abstract

**Background:**

Currently all participants of the Dutch colorectal cancer (CRC) screening program with a positive faecal immunochemical test (FIT) are seen at the outpatient clinic to assess their health status, receive information on colonoscopy and CRC risk, and provide informed consent. However, for many patients this information could probably also safely be exchanged in an online setting, in order to reduce the burden for patients, healthcare system, and environment. In this study we will evaluate if a face-to-face pre-colonoscopy consultation can be replaced by a Digital Intake Tool (DIT) in a CRC screening population.

**Methods:**

This is a prospective multicentre single-arm, non-randomized study with a non-inferiority design. The DIT will triage a total of 1000 participants and inform them about CRC risk, colonoscopy, sedation, and provide bowel preparation instructions. Participants identified as high-risk (i.e., red-triaged) will be contacted by phone or scheduled for an appointment at the outpatient clinic. The primary outcome measure will be adequate bowel preparation rate, defined as the proportion of participants with a Boston Bowel Preparation (BBPS) score ≥ 6. To compare our primary outcome, we will use colonoscopy data from 1000 FIT positive participants who visited the outpatient clinic for pre-colonoscopy consultation. Secondary outcomes will include participation rate, colonoscopy adherence rate, patient experience in terms of satisfaction and anxiety, knowledge transfer, number of outpatient visits that can be averted by the DIT, and cost-effectiveness of the tool. Ethical approval was obtained from the Medical Ethical Committee of the Erasmus Medical Center (MEC-2021-0098).

**Discussion:**

This study aims to assess if a face-to-face pre-colonoscopy consultation can be replaced by an eHealth assessment and education tool in a FIT-based CRC screening program. In case favourable results are established, the intervention evaluated in this study could significantly impact CRC screening programs, benefiting both patients and healthcare systems on a (inter)national scale. Additionally, it would enable more personalized care as the DIT can be easily customized and made feasible in other languages, thereby enhancing healthcare accessibility.

**Trial registration:**

Dutch Trial Register: NL9315, date of registration: March 8th, 2021.

**Supplementary Information:**

The online version contains supplementary material available at 10.1186/s12876-023-03039-0.

## Introduction

### Colorectal cancer screening

Colorectal cancer (CRC) is the third most commonly diagnosed cancer worldwide and the second leading cause of cancer-related mortality [1]. CRC develops from a benign precursor lesion, detection and removal of these polyps prevent CRC, making it suitable for screening by decreasing both incidence and mortality. Therefore, CRC screening is recommended in countries where follow-up and treatment is accessible [2]. Over the past years many countries have introduced organized population-based screening programs, in European countries mostly using faecal immunochemical test (FIT) as the primary screening method [3]. In case of a positive FIT, participants are referred for a face-to-face consultation at the outpatient clinic before colonoscopy is carried out. Aim of this consultation is two-fold: assess participants’ health status and inform participants about CRC risk, colonoscopy including potential complications, sedation and bowel preparation. Both are necessary to achieve a well-informed shared decision.

The increasing burden on outpatient clinics highlights the need for healthcare providers and patients to adopt a more home-based approach to healthcare. Digital health offers a feasible solution, allowing patients to receive care without the need for in-person visits. This not only reduces the burden on the outpatient clinic, but it also offers several benefits for patients as it enables them to receive care at home. In the context of CRC screening, alternatives to the face-to-face pre-colonoscopy consultation for FIT positive participants must be explored. However, it is essential to ensure that these alternatives do not negatively impact important performance indicators of the CRC screening program such as adherence to colonoscopy, bowel cleanliness, or patient experience.

As medical health is undergoing a digital transformation, a lot of research focusses on digital patient education. Bowel preparation is a complex process, and clear instructions are necessary to obtain optimal bowel preparation quality. Currently, a verbal explanation combined with written instructions are standard of care [4]. However, studies have shown that various patient education tools, such as videos and smartphone education, improve bowel preparation and patient satisfaction [5–8]. We can conclude that such education tools could be used before colonoscopy is carried out. However, informing FIT-positives includes more than bowel preparation instructions. Also, information regarding the FIT positive result, CRC risk, and colonoscopy is of high importance and should be provided before colonoscopy is carried out. Before the national CRC screening program was implemented in the Netherlands, Stoop et al. compared face-to-face with telephone pre-colonoscopy consultations in a CRC screening population. They found that colonoscopy adherence and patient satisfaction were lower in the telephone consultation patient group compared to the face-to-face group. The quality of bowel preparation did not differ [9]. Therefore, currently a visit at the outpatient clinic is standard of care for FIT positive participants in the Netherlands.

Besides providing information, FIT-positives’ health status is evaluated during the pre-colonoscopy consultation. Veldhuijzen et al. developed and evaluated a computer-based education pre-colonoscopy tool that is able to triage patients who are referred for colonoscopy. The tool appeared to be equally effective as nurse counselling in terms of bowel preparation, increased adherence to colonoscopy and satisfaction among patients that received the computer-based education modality [10]. However, it is important to note that this randomized controlled trial was performed in a non-screening population, and all included participants had already been triaged and referred for colonoscopy. FIT positive individuals differ from symptomatic patients as they are referred for colonoscopy without having seen a medical professional who has established the participant’s health status and obtained informed consent. Therefore, these results cannot be generalized to a population participating in CRC screening.

Thus far, no data is available on the use of an eHealth tool in a patient population who has not been seen or informed by a medical professional before undergoing an invasive intervention, like a colonoscopy. This study will evaluate the effectiveness and patient experience of a Digital Intake Tool (DIT), that replaces the face-to-face pre-colonoscopy consultation from a hospital setting to home within a FIT-based CRC screening population.

## Methods and analysis

### Aim

The aim of this study is to determine if a face-to-face pre-colonoscopy consultation visit can be replaced by an eHealth tool performed at home in a FIT-based CRC screening program.

### Primary outcome

Adequate bowel preparation is an important quality parameter for colorectal cancer screening programs. Poorly cleaned colons can lead to suboptimal detection of lesions, necessitating repeat colonoscopies. Therefore, the primary outcome of this study is the quality of bowel preparation classified by BBPS. Patient-related factors associated with poor bowel cleaning, for example obstipation, will be collected at baseline.

### Secondary outcomes

Secondary outcomes include participation and response rate, colonoscopy adherence and the number of outpatient visits that are avoided by DIT. The DIT will be evaluated in terms of safety: “Are participants correctly classified as low risk?”, knowledge transfer and usability of the application. Also patient experience will be assessed in terms of satisfaction and anxiety levels both before and after completion of the DIT. Lastly, cost-effectiveness will be assessed.

### Study design

**Fig. 1:**
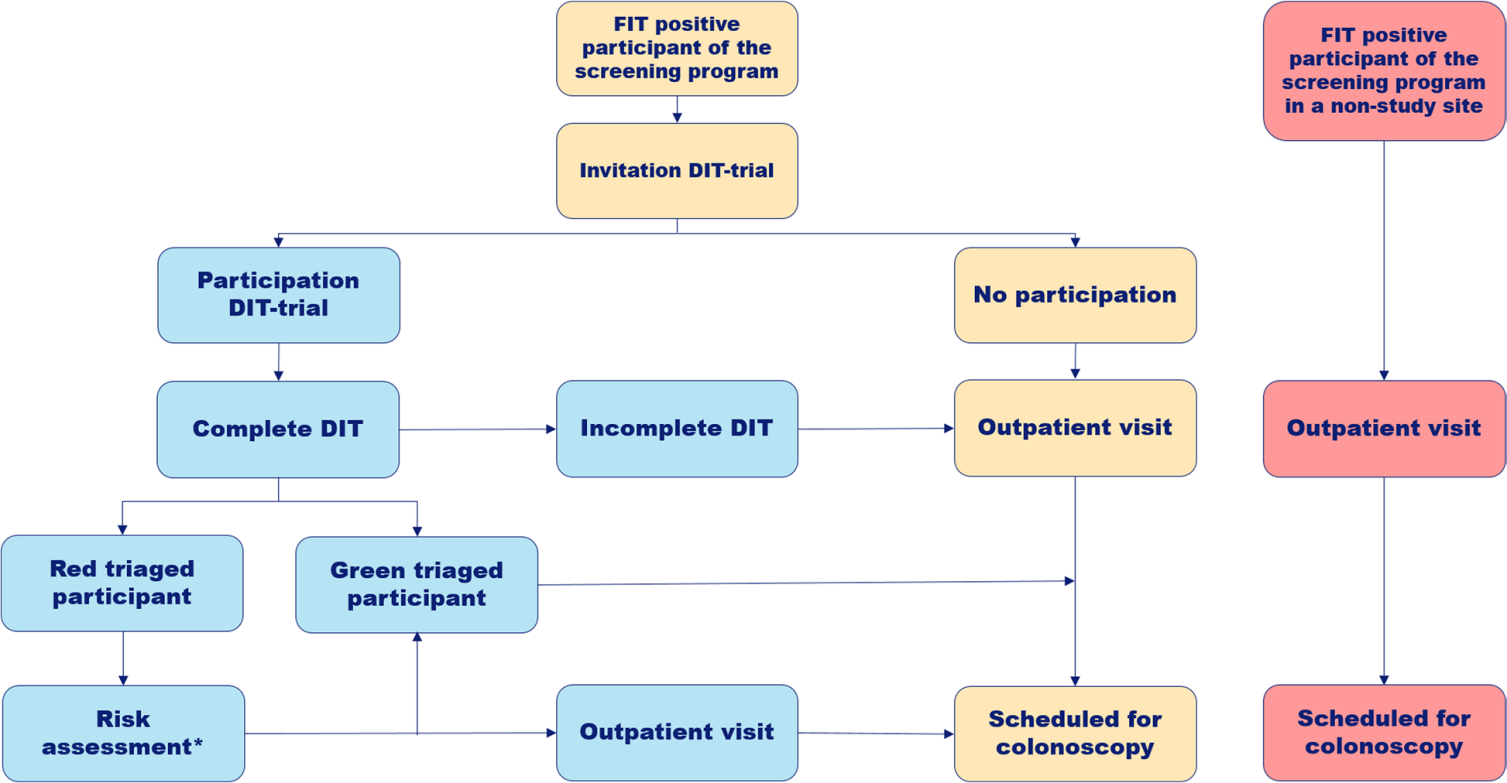
Flowchart of study trial. Study interventions in blue and regular care in yellow, in red the reference cohort. * The intake of red triaged participants will be assessed, and individuals will be contacted via phone or scheduled for an appointment at the outpatient clinic for counselling

This is a prospective, multicentre cohort study with a non-inferiority design to assess the effectiveness of a pre-colonoscopy eHealth tool in participants of a FIT-based CRC screening program. Study sites are located in the Netherlands, and include an academic hospital, regional hospitals, and endoscopy centres. In this single-arm study, all enrolled participants will receive the DIT intervention instead of standard counselling at the outpatient clinic. For analyses of our primary outcome, we will compare colonoscopy data of the study participants with that of 1000 FIT positive participants who received pre-colonoscopy counselling at the outpatient clinic of endoscopy centres that did not participate in the DIT-trial (Fig. 1). Additionally, we will assess patient-related outcome measures, including anxiety levels, satisfaction and knowledge transfer among a reference cohort consisting of 100 FIT-positive participants who received pre-colonoscopy counselling at the outpatient clinic. This study was designed according to the Standard Protocol Items: Recommendations for Interventional Trials (SPIRIT) 2013 shown in Supplementary Table 1.

### Study setting: Dutch CRC screening program

In the Netherlands, a national CRC screening program was gradually implemented in 2014, using biennial FIT. All individuals between the age of 55 and 75 years old receive an information leaflet with information on CRC, FIT, advantages and disadvantages of CRC screening enclosed with a FIT. Participation and returning FIT is free of charge. In case of a positive FIT, colonoscopy is recommended. FIT positive participants receive, by mail, the result of their stool test together with an invitation for a pre-colonoscopy consultation at the outpatient clinic of a certified endoscopy centre. Once both the medical professional and the patient have agreed to proceed with screening colonoscopy, detailed information on bowel preparation is provided during the pre-colonoscopy consultation, and colonoscopy is scheduled within a three-week timeframe. Reasons not to schedule screening colonoscopy are a life expectancy of less than five years and FIT positive individuals who are unable or unwilling to undergo colonoscopy [11]. Each year around 2.2 million individuals are invited to participate in the national CRC screening program, approximately 70% return their FIT of which 4–5% have a positive FIT. As a consequence, approximately 70.000 participants are referred to the outpatient clinic annually [12].

### Sample selection

All FIT-positive participants of the Dutch CRC screening program are eligible for inclusion if they are able to provide informed consent. Patients are excluded when having 1) a visual disability 2) lack of internet access or do not have a relative with internet access 3) Dutch illiteracy. FIT-positives are requested to contact the outpatient clinic of the endoscopy centre they have been referred to, prior to their scheduled face-to-face consultation. Patients will be recruited during this phone contact. If they choose to have a face-to-face visit instead of the DIT intervention, they will not be included in the study.

### Intervention

**Fig. 2:**
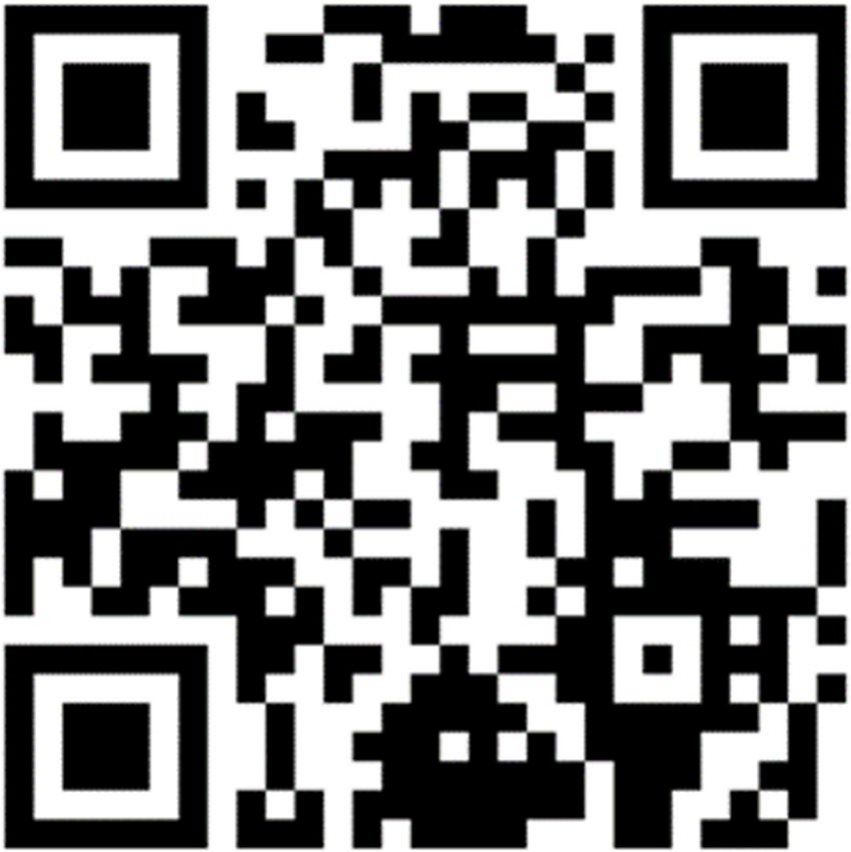
QR code to animation video. To access a preview of the animation videos used in the DIT, scan the QR code

All enrolled participants will receive the DIT instead of a counselling visit. The DIT is a web-based platform with 3D animation videos guided by a voiceover and a medical questionnaire. The patient education in the DIT was developed in collaboration with Informed. B.V., which is a company specialized in developing animation tools to inform patients, including low literacy, about medical procedures. To ensure that the information provided met the national requirements, the Dutch National Institute for Public Health and Environment (RIVM) as well as the Dutch screening organization were consulted. The animation videos provide information about the positive FIT result, CRC risk and colonoscopy procedure, including information about the complication risks and sedation. For a preview of the animation videos, scan the QR-code shown in Fig. 2. Once participants complete the DIT, a risk assessment will be conducted, categorizing participants’ intake result as “green” in case no further action is required or “red” if action is necessary. In case the latter, a gastroenterology nurse or gastroenterologist will evaluate the participant on paper before proceeding with colonoscopy, and determine if an outpatient consultation is necessary for further evaluation. All participants will be contacted by phone to schedule the colonoscopy, and personalized animated bowel preparation instructions will be send via the DIT.

### Patient and public involvement

Study inclusion has been ongoing since October 2021 and has an anticipated end date of March 2024. The first ten included participants who used the DIT were interviewed to evaluate its usability. No technical issues were identified, only minor modifications were suggested, such as rephrasing certain questions of the medical questionnaire and a navigation tool to allow for easy movement between the pages. Moreover, gastroenterologists and gastroenterology nurses were involved in the first evaluation of the DIT and asked to provide feedback. The provided feedback from a patient and healthcare perspective regarding usability of the DIT was evaluated and implemented for future users.

### Sample size calculation

A sample size calculation was performed for demonstrating non-inferiority of the DIT versus all participants visiting the outpatient clinic with regard to the primary endpoint; adequate bowel preparation, defined as a Boston Bowel Preparation Score (BBPS) of 6 or higher. The choice of non-inferiority design was motivated by the expectation that the DIT will improve secondary outcomes regarding costs and satisfaction. According to the Dutch national CRC screening organization at least 90% of all participants in the CRC screening program must have a BBPS of at least 6. Based on earlier data, currently in 97.5% a BBPS of 6 or higher is achieved among participants of the Dutch screening program. With a clinically acceptable difference of -0.5%, corresponding to at least 89.5% of participants achieving adequate bowel preparation, the non-inferiority margin is set at -8.0%. To achieve 90% power with a one-sided Exact test at a 2.5. significance level, 738 participants are needed. Anticipating 30% loss to follow-up or withdrawal of participants, we aim to include 1000 participants.

### Participation rate

In order to evaluate the DIT in a CRC screening population, participation and response rate are important outcome measurements. The participation rate will be calculated by dividing the number of included participants by the total number of eligible individuals who are approached. In addition, all individuals who decline to participate will be asked about the reason for their preference for a face-to-face consultation over the DIT intervention. Also age and sex of non-participants will be collected. The response rate will be calculated by dividing the number of participants who completed the DIT by the number of included participants.

### Measurements of patient experience

#### Knowledge transfer

To assess knowledge transfer, a CRC screening specific questionnaire was developed. The knowledge questionnaire of Denters et al., developed earlier to assess informed decisions in a FIT-based CRC screening pilot study, was adjusted to the current CRC screening strategy [13]. Several questions were added and irrelevant questions were removed based on the required topics stated by the Dutch National Institute for Health and Environment (RIVM) regarding screening information that is provided [14]. Items were selected to cover the following knowledge domains: CRC in general, CRC screening specific (e.g., risk of having CRC), colonoscopy and bowel preparations. In total, 16 items were formulated as statements that can be answered with ‘true’ or ‘false’. Each correct answer given by the participant will be awarded with one point, with a maximum score of 16. After participants undergo screening colonoscopy, they will be asked (using an 11-point Likert scale) if their expectations regarding colonoscopy preparations and the colonoscopy procedure were met.

The knowledge transfer questionnaire will be validated among the first 150 participants who will receive the DIT. The validation process is beyond the scope of this study; however, it will ensure that the questionnaire effectively measures the domains, and will allow us to establish a minimum score that indicates an adequate level of knowledge.

#### Anxiety

The shortened version of the Spielberger State-Trait Anxiety Inventory (STAI-6) will be used to measure a change in patients’ state of anxiety regarding colonoscopy after receiving all information via animations in the DIT [15]. Participants will be requested to fill in STAI-6 both at the beginning and upon completion of the DIT. Furthermore, specific questions will be asked to assess a change in CRC screening specific worries. A detailed description of the questionnaire can be found in Table 1.

**Table 1:**
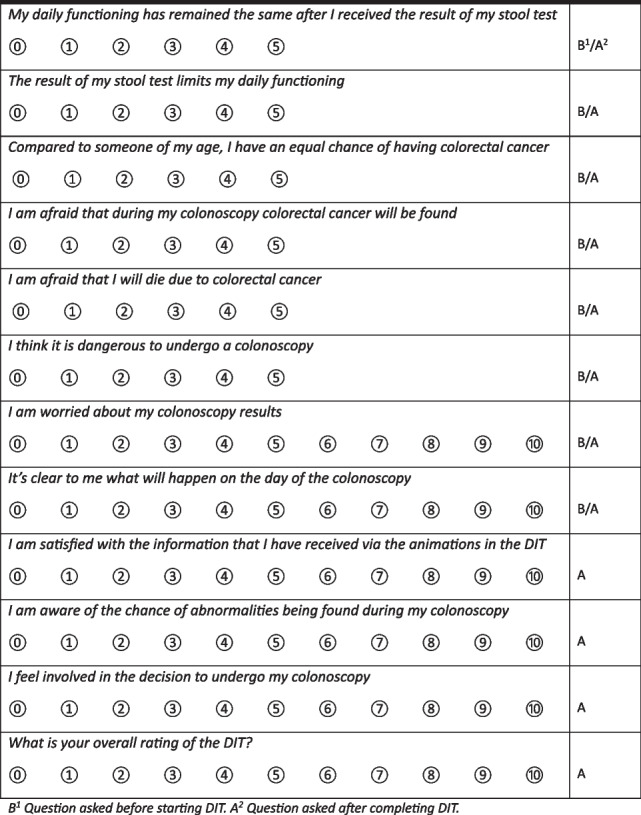
Questions about patient experiences regarding the DIT

#### Satisfaction

**Fig. 3:**
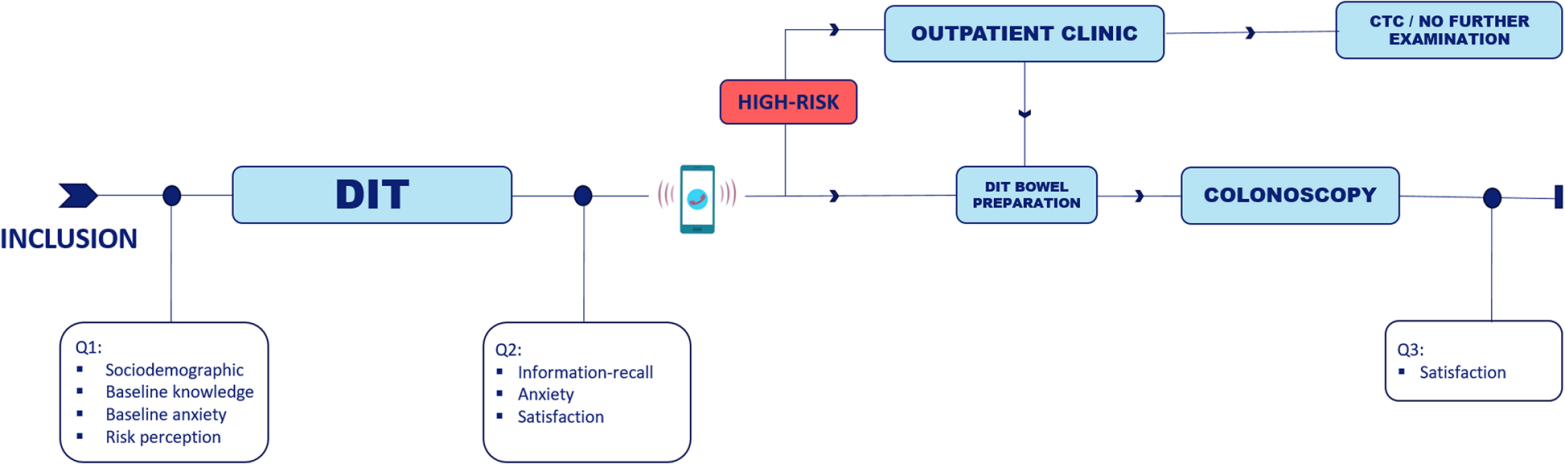
Flowchart illustrating time points for participants. DIT, Digital Intake Tool, Q, Questionnaire. CTC, CT-colonography. If patient and/or gastroenterologist choose not to pursue for additional diagnostic testing, no further examinations will be performed﻿

For patient satisfaction, four measures will be included in the questionnaire. “How satisfied are you with the received information”, “What is your overall rating of the DIT?”, “Would you recommend the DIT to your peers?” and “If you are referred for another screening colonoscopy, which type of consultation would you prefer? A face-to-face consultation, an eHealth tool like the DIT, counselling via phone or counselling via a video call”. Also motives of choice and points of improvement will be evaluated. To verify participants’ satisfaction, participants will not only be requested to complete the questionnaires after completing the DIT, but also after receiving colonoscopy (Fig. 3).

### Data collection

Demographic data and patient experiences will be collected via the DIT and will be stored in the electronic data management system, Castor Electronic Data Capture. Each participant will receive a unique study number after study enrolment. This study number will be used for all study documentation. Colonoscopy data will be reported by the gastroenterologist who performed the colonoscopy. This data is stored in the national database of the Dutch screening organization, Screen-IT. By linkage to Screen-IT we will retrieve the following colonoscopy data: BBPS, caecal intubation, reason for incomplete colonoscopy, American Society of Anaesthesiologists physical status classification (ASA), Gloucester Comfort Score and re-colonoscopy due to inadequate bowel preparation or incomplete colonoscopy. In addition, colonoscopy data will be retrieved from 1000 FIT-positive participants of the CRC screening program who did not participate in the DIT-trial. This will allow us to compare the DIT intervention to face-to-face consultation at the outpatient clinic with regard to our primary outcome.

### Statistical analysis

Continuous data will be summarized using means and standard deviations, or medians and interquartile ranges in case of skewed distributions. Categorical data will be presented in relative frequencies and counts. Comparison of normally distributed continuous variables will be done with the Student t-test or Mann-Whitney U test in case of skewed distributions. Categorical outcomes will be analysed using χ2 test. Non-inferiority of our primary outcome will be evaluated by whether the lower bound of the two sided 95% confidence interval of the observed difference in adequate bowel preparation rate is above the non-inferiority margin of 0.5%.

For repeated measurements within participants, e.g. the total STAI-6 score before and after the DIT, paired tests will be used to analyse differences. Differences are considered significant in case *p* ≤ 0.05. Other secondary outcome parameters will only be obtained from study participants who underwent the DIT intervention, these results will be reported using descriptive statistics.

Statistical analysis will be performed using IBM SPSS statistics.

## Discussion

This study will assess the effectiveness and patient experience of an eHealth assessment and education tool that replaces pre-colonoscopy consultation from a hospital setting to home within a FIT based CRC screening program. The intervention of this study has the potential to reduce the societal burden of CRC screening. Favourable results could result in the introduction of an easy and equally effective tool for participants who are being evaluated for screening colonoscopy as it can be done at home at any suitable time. This would be beneficial for the public, as a considerable number of participants referred for colonoscopy are employed and would not have to take time off work. Moreover, it will facilitate healthcare organizations as the capacity of the gastroenterology outpatient departments can be used more effectively. Firstly, with fewer screening participants occupying available outpatient appointments, there will be an increased availability to accommodate other patients. Secondly, if the DIT is less time-consuming compared to a face-to-face consultation, it will save valuable time for medical professionals. This would enable them to see other patients. The COVID-19 pandemic emphasizes the need to develop innovations that shift healthcare to a more home-based setting. Also, implementation of the DIT could potentially reduce healthcare costs. In case the DIT is able to safely and effectively triage and inform all ASA I and half of the ASA II FIT positive participants 50% of the outpatient visits will be averted in the Netherlands. The results of this study could also be of interest for other outpatient consultations that are currently done before an (invasive) intervention is carried out, for example a total knee replacement which is a regular straightforward surgical procedure.

Furthermore, the DIT could be useful for regions with low CRC screening participation rates, as it has the potential to increase colonoscopy adherence. Although European guidelines require adherence to follow-up colonoscopy to be above 90% to achieve screening benefits, this is not the case in the majority of the population-based CRC screening programs [16]. As the participation and adherence rates for CRC screening in the Netherlands are already among the highest in the world, this would be of even greater significance to other countries. A meta-analysis evaluating adherence to colonoscopy following a positive stool test estimated adherence at 72.5% and a study in the United States showed that only 60% completed follow-up after a positive initial screening test [17, 18]. Several patient and environmental factors have been associated with non-adherence, including living in remote areas with long driving times to healthcare facilities, lack of knowledge on CRC screening and low perceived risk of developing CRC [19–21]. The DIT offers solutions to these barriers. Firstly, the pre-colonoscopy consultation can be done at home. Secondly, the communication strategy of the DIT might enhance participants’ knowledge on screening and CRC risk. Providing medical information via spoken animation is one of the best ways to communicate complex health information, especially to patients with low (health) literacy, and in a CRC screening setting ethnic minorities have indicated that verbal and visual information is the best communication strategy [22, 23]. Therefore, the DIT has the potential to overcome the aforementioned barriers and may lead to a well-informed decision with the potential to increase colonoscopy adherence in regions with low adherence rates.

It is known that individuals from ethnic minority groups are difficult to reach, especially in a CRC screening setting [24, 25]. Many of the ethnic minority groups living in the Netherlands have a poor understanding of the Dutch language and lower (health) literacy. Therefore, a limitation of this study is that non-Dutch speakers are excluded. For participation in studies, patients need to be able to read and understand the patient information form in Dutch and sign for informed consent to participate in the DIT-trial. However, in case the study shows favourable results, the DIT can easily be adapted to support additional languages. Follow-up studies should still be carried out to validate the DIT among non-Dutch speakers. However, in addition to language barriers, ethnic minority groups often experience a lower socioeconomic status (SES) [24]. In this study we will be able to evaluate the DIT among participants from different socioeconomic backgrounds, low SES serving as an approach to ethnic minority groups, based on postal code. A second limitation is the recruitment process of the study. Because of the non-randomized study design, selection bias may be present. To stay in line with the current logistics and regulations of the Dutch CRC screening program, we are unfortunately unable to conduct a randomized controlled trial. Consequently, it is possible that only individuals who prefer eHealth over regular care will be included. Nevertheless, we will collect all reasons given by non-participants refraining from participation. This will help to gain more insight into the public’s attitude and preferences towards digitalization of this process.

In conclusion, in this study we will evaluate if pre-colonoscopy outpatient visits can be replaced by an eHealth tool for FIT positive participants of the Dutch CRC screening program.

### Supplementary Information

**Additional file 1: Supplementary table 1. **SPIRIT 2013 Checklist: Recommended items to address in a clinical trial protocol.

## Data Availability

The dataset generated during this study will not be publicly available due to General Data Protection Regulation, but will be available from the corresponding author on reasonable request.

## Abbreviations

ASA: American Society of Anaesthesiologists physical status classification
BBPS: Boston Bowel Preparation
CRC: colorectal cancer
DIT: Digital Intake Tool
FIT: faecal immunochemical test
RIVM: Dutch National institute for Public Health and Environment
STAI: Spielberger State-Trait Anxiety Inventory
SES: socioeconomic status
SPIRIT: Recommendations for Interventional Trials
TKI-LSH: Top Sector Life Sciences & Health

## References

[1] Sung H, Ferlay J, Siegel RL, Laversanne M, Soerjomataram I, Jemal A (2021). Global Cancer statistics 2020: GLOBOCAN estimates of incidence and mortality worldwide for 36 cancers in 185 countries. CA Cancer J Clin..

[2] Lauby-Secretan B, Vilahur N, Bianchini F, Guha N, Straif K (2018). International Agency for Research on Cancer handbook working G. The IARC perspective on colorectal Cancer screening. N Engl J Med..

[3] Cardoso R, Guo F, Heisser T, Hoffmeister M, Brenner H. Utilisation of colorectal Cancer screening tests in European countries by type of screening offer: results from the European health interview survey. Cancers (Basel). 2020;12(6) 10.3390/cancers12061409 PMC7352919 32486077

[4] Hassan C, East J, Radaelli F, Spada C, Benamouzig R, Bisschops R (2019). Bowel preparation for colonoscopy: European Society of Gastrointestinal Endoscopy (ESGE) guideline - update 2019. Endoscopy..

[5] Li P, He X, Yang X, Du J, Wu W, Tu J (2022). Patient education by smartphones for bowel preparation before colonoscopy. J Gastroenterol Hepatol..

[6] Veldhuijzen G, Klemt-Kropp M, Noomen C, Van Esch AA, Tjwa ET, Drenth J (2017). Computer-assisted instruction before colonoscopy is as effective as nurse counselling, a clinical pilot trial. Endosc Int Open..

[7] Shaw MJ, Beebe TJ, Tomshine PA, Adlis SA, Cass OW (2001). A randomized, controlled trial of interactive, multimedia software for patient colonoscopy education. J Clin Gastroenterol..

[8] Park JS, Kim MS, Kim H, Kim SI, Shin CH, Lee HJ (2016). A randomized controlled trial of an educational video to improve quality of bowel preparation for colonoscopy. BMC Gastroenterol..

[9] Stoop EM, de Wijkerslooth TR, Bossuyt PM, Stoker J, Fockens P, Kuipers EJ (2012). Face-to-face vs telephone pre-colonoscopy consultation in colorectal cancer screening; a randomised trial. Br J Cancer..

[10] Veldhuijzen G, Klemt-Kropp M, Terhaar Sive Droste JS, van Balkom B, van Esch AAJ, Drenth JPH (2021). Computer-based patient education is non-inferior to nurse counselling prior to colonoscopy: a multicenter randomized controlled trial. Endoscopy..

[11] The National Institute for Public Health and the Environment (RIVM). Framework for the Execution of the Dutch Colorectal Cancer Screening Programme, 2021. Available from: https://www.rivm.nl/documenten/framework-execution-dutch-crc-screening-2021-0. Accessed Dec 2020.

[12] The National Institute for Public Health and the Environment (RIVM). National Monitoring of the Colorectal Cancer Screening Programme in the Netherlands, 2021. Available from: https://www.rivm.nl/en/documenten/monitor-colorectal-cancer-2021. Accessed 4 Oct 2022.

[13] Denters MJ, Deutekom M, Essink-Bot ML, Bossuyt PM, Fockens P, Dekker E (2015). Assessing knowledge and attitudes towards screening among users of Faecal immunochemical test (FIT). Health Expect..

[14] The National Institute for Public Health and the Environment (RIVM). Meedoen aan bevolkingsonderzoeken en screeningen. Available from: https://www.rivm.nl/bevolkingsonderzoeken-en-screeningen/meedoen.

[15] Marteau TM, Bekker H (1992). The development of a six-item short-form of the state scale of the Spielberger state-trait anxiety inventory (STAI). Br J Clin Psychol..

[16] Moss S, Ancelle-Park R, Brenner H (2012). International Agency for Research on C. European guidelines for quality assurance in colorectal cancer screening and diagnosis. First Edition--Evaluation and interpretation of screening outcomes. Endoscopy..

[17] Gingold-Belfer R, Leibovitzh H, Boltin D, Issa N, Tsadok Perets T, Dickman R (2019). The compliance rate for the second diagnostic evaluation after a positive fecal occult blood test: a systematic review and meta-analysis. United Europ Gastroenterol J..

[18] Etzioni DA, Yano EM, Rubenstein LV, Lee ML, Ko CY, Brook RH (2006). Measuring the quality of colorectal cancer screening: the importance of follow-up. Dis Colon Rectum..

[19] Morris S, Baio G, Kendall E, von Wagner C, Wardle J, Atkin W (2012). Socioeconomic variation in uptake of colonoscopy following a positive faecal occult blood test result: a retrospective analysis of the NHS bowel Cancer screening Programme. Br J Cancer..

[20] Bie AKL, Brodersen J (2018). Why do some participants in colorectal cancer screening choose not to undergo colonoscopy following a positive test result? A qualitative study. Scand J Prim Health Care..

[21] Llovet D, Serenity M, Conn LG, Bravo CA, McCurdy BR, Dubé C (2018). Reasons for lack of follow-up colonoscopy among persons with a positive fecal occult blood test result: a qualitative study. Am J Gastroenterol..

[22] Woudstra AJ, Dekker E, Essink-Bot ML, Suurmond J (2016). Knowledge, attitudes and beliefs regarding colorectal cancer screening among ethnic minority groups in the Netherlands - a qualitative study. Health Expect..

[23] Meppelink CS, van Weert JC, Haven CJ, Smit EG (2015). The effectiveness of health animations in audiences with different health literacy levels: an experimental study. J Med Internet Res..

[24] Deutekom M, van Rijn AF, Dekker E, Blaauwgeers H, Stronks K, Fockens P (2009). Uptake of faecal occult blood test colorectal cancer screening by different ethnic groups in the Netherlands. Eur J Pub Health..

[25] Genoff MC, Zaballa A, Gany F, Gonzalez J, Ramirez J, Jewell ST (2016). Navigating language barriers: a systematic review of patient Navigators' impact on Cancer screening for limited English proficient patients. J Gen Intern Med..

