# Correction: A digital intake tool to avert outpatient visits in a FIT-based colorectal cancer screening population: study protocol of a multicentre, prospective non-randomized trial - the DIT-trial

**DOI:** 10.1186/s12876-024-03278-9

**Published:** 2024-06-19

**Authors:** Fleur E. Marijnissen, Pieter J. F. de Jonge, Nicole S. Erler, Sohal Y. Ismail, Iris Lansdorp-Vogelaar, Manon C. W. Spaander

**Affiliations:** 1 Department of Gastroenterology and Hepatology, Erasmus MC, University Medical Center, Rotterdam, the Netherlands; 2 Department of Biostatistics, Erasmus MC, University Medical Center, Rotterdam, the Netherlands; 3 Department of Epidemiology, Erasmus MC, University Medical Center, Rotterdam, the Netherlands; 4 Department of Psychiatry, Section Medical Psychology and Psychotherapy, Erasmus MC, University Medical Center, Rotterdam, the Netherlands; 5 Department of Public Health, Erasmus MC, University Medical Center, Rotterdam, the Netherlands

**Correction: BMC Gastroenterol 24, 38 (2024).**

10.1186/s12876-023-03039-0

Following publication of the original article, it was identified that there was an error in reporting the sample size calculation. The non-inferiority margin was inaccurately reported as -0.5%, whereas it should be correctly stated as -8.0%. The stated − 0.5% refers to the clinically acceptable difference in the context of the national screening program guideline, which require that a minimum of 90% of the participants achieve adequate bowel preparation. Given this clinically acceptable difference of -0.5%, corresponding to at least 89.5% of the participants achieving adequate bowel preparation, and the current adequate bowel preparation rate of 97.5%, the correct non-inferiority margin is -8.0%.

Importantly, the error is only in the wording of the sample size description. There is no alteration to the total number of inclusions, as the calculation was based on the correct non-inferiority margin of 0.08. Therefore, the integrity of the study’s design and its conclusions remain intact.

The original article has been updated.

